# High activity and selectivity of single palladium atom for oxygen hydrogenation to H_2_O_2_

**DOI:** 10.1038/s41467-022-32450-6

**Published:** 2022-08-12

**Authors:** Shiming Yu, Xing Cheng, Yueshuai Wang, Bo Xiao, Yiran Xing, Jun Ren, Yue Lu, Hongyi Li, Chunqiang Zhuang, Ge Chen

**Affiliations:** 1 Beijing Key Laboratory for Green Catalysis and Separation, Faculty of Environment and Life, Beijing University of Technology, Beijing, 100124 P. R. China; 2 Faculty of Materials and Manufacturing, Beijing University of Technology, Beijing, 100124 P. R. China; 3 North University of China, Taiyuan, 030051 P. R. China

**Keywords:** Environmental monitoring, Heterogeneous catalysis, Catalytic mechanisms

## Abstract

Nanosized palladium (Pd)-based catalysts are widely used in the direct hydrogen peroxide (H_2_O_2_) synthesis from H_2_ and O_2_, while its selectivity and yield remain inferior because of the O-O bond cleavage from both the reactant O_2_ and the produced H_2_O_2_, which is assumed to have originated from various O_2_ adsorption configurations on the Pd nanoparticles. Herein, single Pd atom catalyst with high activity and selectivity is reported. Density functional theory calculations certify that the O-O bond breaking is significantly inhibited on the single Pd atom and the O_2_ is easier to be activated to form *OOH, which is a key intermediate for H_2_O_2_ synthesis; in addition, H_2_O_2_ degradation is shut down. Here, we show single Pd atom catalyst displays a remarkable H_2_O_2_ yield of 115 mol/g_Pd_/h and H_2_O_2_ selectivity higher than 99%; while the concentration of H_2_O_2_ reaches 1.07 wt.% in a batch.

## Abstract (web summary)

Nanosized Pd-based catalysts are widely used in the direct hydrogen peroxide (H_2_O_2_) synthesis from H_2_ and O_2_, while the selectivity and yield of H_2_O_2_ remain inferior. Here, a remarkable H_2_O_2_ yield of 115 mol/g_Pd_/h and H_2_O_2_ selectivity higher than 99% are reported using a Pd single-atom catalyst for the direct synthesis of H_2_O_2_.

## Introduction

**Fig. 1:**
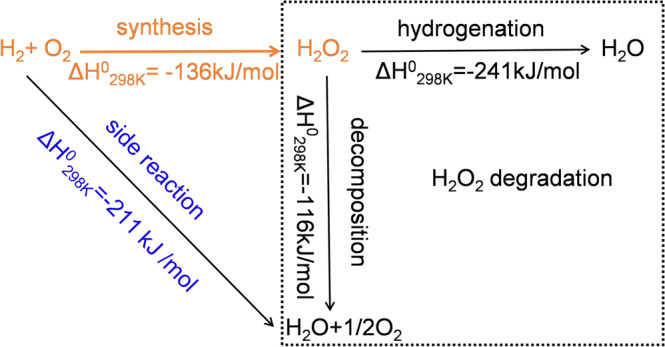
Schematic illustration in the direct synthesis of H_2_O_2_. all reactions in the direct synthesis of H_2_O_2_.

Hydrogen peroxide (H_2_O_2_) is one of the most important chemicals in industry, used in the production of fine chemicals and medicine, rocket fuels, sterilization, bleaching, and so on^1,2^. In the conventional process, H_2_O_2_ is mainly produced via the anthraquinone method, which consists of hydrogenation and oxidation of anthraquinone successively. The quest for an ecofriendly process for H_2_O_2_ synthesis is driven by the current disadvantages including high energy consumption and heavy pollution^3,4^. Under such circumstances, the direct synthesis of H_2_O_2_ from hydrogen (H_2_) and oxygen (O_2_) is an efficient and clean strategy to replace the anthraquinone oxidation process^5^. However, this process is challenging because of many parallel and consecutive reactions, as shown in Fig. 1. Specifically, compared to the synthesis of H_2_O_2_, it is thermodynamically more favorable to the production of H_2_O via breaking O–O bonds, while the generated H_2_O_2_ also degrade through further hydrogenation and decomposition^6,7^.

Palladium (Pd)^8,9^ is a widely used catalyst in the direct synthesis of H_2_O_2_ due to its excellent hydrogenation activity. However, Pd is also active for side reactions and subsequent H_2_O_2_ degradation^10,11^, resulting in an inferior H_2_O_2_ selectivity and poor yield. Pd-based nanoalloy catalyst (e.g., Pd-Pt, Pd-Au, Pd-Zn, Pd-Ag, Pd-Te, Pd-Sb, Pd-Sn)^5,12–23^ can effectively modify the electronic structure of Pd, thus inhibiting side reactions and H_2_O_2_ degradation. Besides, H_2_O_2_ can also be stabilized by adding strong acids or halides to the solvent, while it will cause metal shedding and needs a subsequent purification process to obtain pure H_2_O_2_^24,25^. Therefore, the rational design of a catalyst with high activity, high selectivity for oxygen hydrogenation to H_2_O_2_ as well as low degradation towards the generated H_2_O_2_ remains a formidable challenge.

The selectivity toward H_2_O_2_ is mainly determined by the competitive reactions between *OOH formation and O–O bond cleavage on catalysts which highly depend on the O_2_ adsorption configuration^18,26–32^. The Pd nanoparticles involve various adsorption modes such as “side-on”, “end-on” and “bridge”, while the adsorption of O_2_ on isolated Pd atom is usually the “end-on” type and could therefore reduce the possibility of O–O bond breaking. Thus, it would be encouraging to develop a single Pd atom catalyst to improve selectivity towards H_2_O_2_.

In this work, we have prepared a series of catalysts, among which the single Pd atom catalyst displays a remarkable H_2_O_2_ yield of 115 mol/g_Pd_/h and a selectivity higher than 99%, surpassing the performance of reported Pd-based catalysts. Besides, H_2_O_2_ degradation is also shut down, making it an ideal catalyst. The concentration of H_2_O_2_ reaches 1.07 wt.% in a batch. Density functional theory calculations reveal that the high yield and selectivity is believed to have originated from the high energy barrier of both O–O bond dissociation and H_2_O_2_ dissociation on the single Pd atom catalyst.

## Results

### Synthesis and characterization of materials

**Fig. 2:**
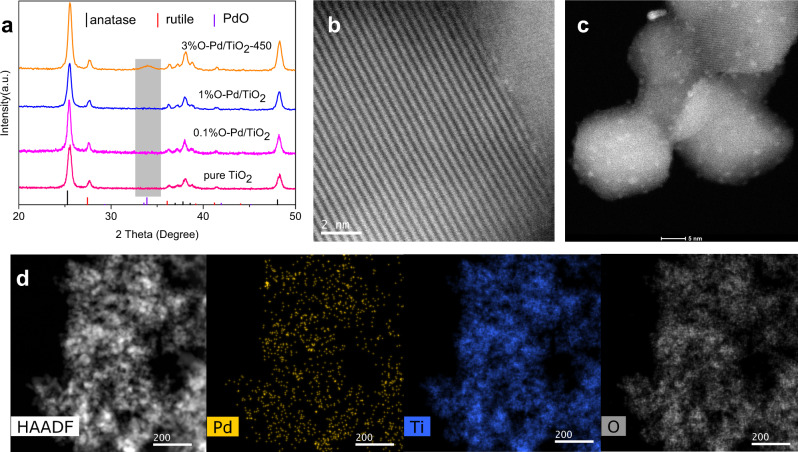
Structure characterization of the catalysts. **a** XRD patterns of O-Pd/TiO_2_. **b** HAADF-STEM images of 0.1%O-Pd/TiO_2_ with 2 nm scale bars. **c** HAADF-STEM images of 1% O-Pd/TiO_2_ with 5 nm scale bars. **d** STEM-EDS elemental mappings 0.1%O-Pd/TiO_2_, scale bars 200 nm.

A series of TiO_2_ (commercial P25)-supported O-Pd (oxidized Pd) catalysts were successfully synthesized through a simple hydrothermal method (Details in methods). For comparison, M-Pd (metallic Pd)/TiO_2_ catalysts were also synthesized. Catalysts are named after the Pd loading. In the XRD pattern of O-Pd/TiO_2_ samples (Fig. 2a and Supplementary Fig. 1a), there are no peaks associated with Pd species observed for 0.1% O-Pd/TiO_2_ and 1% O-Pd/TiO_2_, while a broad diffraction peak of PdO (101) around 34° appeared in 3% O-Pd/TiO_2_ XRD pattern. In contrast, the M-Pd/TiO_2_ samples showed diffraction peaks of Pd (111) and Pd (200) around 40° and 46° except for 0.1%M-Pd /TiO_2_ (Supplementary Fig. 1b), and the intensity of the peaks increased with the increase of palladium loading. XRD refinements results show that the lattice parameters of TiO_2_ between samples are almost identical (Supplementary Fig. 1c–f), so the effect of different loading on the lattice of TiO_2_ could be excluded. The catalyst morphology was further investigated by transmission electron microscopy (TEM) and aberration-corrected transmission electron microscopy (AC-TEM). No obvious Pd species can be seen from the TEM image of 0.1% O-Pd/TiO_2_ (Supplementary Fig. 2a); however, single Pd atoms can be seen distributed over the TiO_2_ by using AC-TEM (Fig. 2b). Because of the low *z*-contrast between Pd (*z* = 46) and Ti (*z* = 22), the single Pd atom in Fig. 2b may not be very clear. When the Pd loading reaches 1%, clusters were observed on the TiO_2_, and the sizes are about 2 nm (Fig. 2c and Supplementary Fig. 2b). EDS mapping provided further evidence for a homogeneous distribution of Pd over the catalyst (Fig. 2d).

**Fig. 3:**
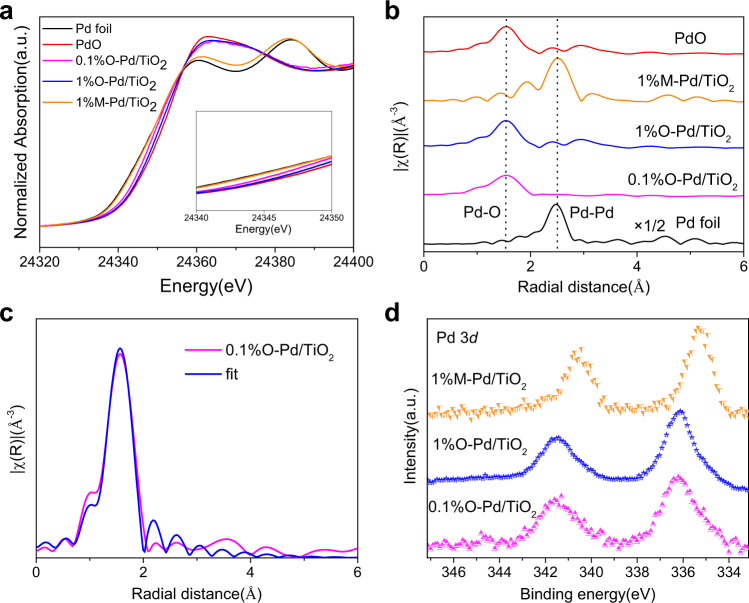
Spectroscopic characterization. **a** XANES spectra at the Pd K-edge and **b** The Fourier transforms of Pd K-edge EXAFS spectra for catalysts, PdO, and Pd foil. **c** The FT EXAFS fitting spectrum of 0.1% O-Pd/TiO_2_ at R-space. **d** The X-ray photoelectron spectra of catalysts.

X-ray absorption spectroscopy (XAS) measurements were performed to probe the local environment of Pd atoms in O-Pd/TiO_2_ and M-Pd/TiO_2_. The X-ray absorption near edge structure (XANES) region of the XAS spectrum provides information about the oxidation state of Pd. The Pd K-edge absorption edge position for O-Pd/TiO_2_ were like that of standard PdO sample (Fig. 3a), they were at higher photon energies than Pd metal, indicating that the Pd atoms in O-Pd/TiO_2_ were in the oxidation state. Fourier transformed R-space curves of the Pd K-edge EXAFS spectra revealed the bonding environment of Pd species in O-Pd/TiO_2_, an obvious peak at 1.75 Å was observed in the R-space spectrum of samples which was believed to be a Pd-O bond (Fig. 3b). The Pd metal foil and 1% M-Pd/TiO_2_ showed an intense Pd-Pd feature at around 2.5 Å, which was absent in the O-Pd/TiO_2_ samples (Fig. 3b), confirming that no Pd-Pd bonds were present in the O-Pd/TiO_2_. And the fitting results showed the coordination number of Pd-O is 4 for 0.1% O-Pd/TiO_2_ (Fig. 3c and Supplementary Table 1), representative of the oxidized Pd single atoms on the surface of TiO_2_^33,34^. These results confirmed that the Pd was atomically dispersed on TiO_2_, which was consistent with the results of AC-TEM. The Pd K-edge absorption edge position and Fourier transformed R-space curves for M-Pd/TiO_2_ were like Pd foil, indicating that the metallic Pd states in M-Pd/TiO_2_ samples (Fig. 3a and Supplementary Fig. 3). X-ray photoelectron spectroscopy (XPS) analyses were presented in Fig. 3d, there is a strong signal of Pd^2+^ species (336.3 eV) in the spectra of 0.1% and 1% O-Pd/TiO_2_. For M-Pd/TiO_2_, we find that there is a strong signal of Pd^0^ (334.9 eV) (Fig. 3d and Supplementary Fig. 4a). The absence of Cl peaks in the XPS spectra indicates that most of the chloride ions have been removed during the washing process, so the influence of chlorine ions on catalytic performance is excluded. (Supplementary Fig. 4b, c).

#### Catalytic performance

The O_2_ hydrogenation to H_2_O_2_ was performed in a batch reactor with temperature at 2 °C controlled by an ice bath. We show that the catalytic performance of Pd catalysts in oxidation state (O-Pd/TiO_2_) (including single Pd atom (0.1%), Pd cluster (1%)) are all better than that of metallic Pd catalyst (M-Pd/TiO_2_). (Table 1, Entry 1–11). Particularly, the single Pd atom catalytic performance was up to 115 mol/kg_cat_/h, and the selectivity of H_2_O_2_ was more than 99% (Table 1, Entry 1–2), which is superior to all other samples as well as the reported state-of-the-art catalysts (Table 1, Entry 1–2, 3–20). The Pd cluster catalyst (1% O-Pd/TiO_2_) has a H_2_O_2_ yield of 79 mol/kg_cat_/h, while the selectivity is 58% (Table 1, Entry 3–5), and the PdO nanoparticles demonstrate only a 7% selectivity to H_2_O_2_. For M-Pd/TiO_2_, the H_2_O_2_ yield are much lower than that of O-Pd/TiO_2_ (Table 1, Entry 8-11). For example, the H_2_O_2_ yield of 3% and 5% M-Pd/TiO_2_ were 46 and 42 mol/kg_cat_/h, respectively. Interestingly, the catalytic activity of 0.1%M-Pd/TiO_2_ is very limited (Table 1, Entry8), as H_2_O_2_ yield was not detected. Also, we have synthesized 9% Pd/C catalyst, and the activity and selectivity of Pd/C catalyst was inferior to both O-Pd/TiO_2_ and M-Pd/TiO_2_ catalysts. (Table 1, Entry 12).

**Table 1:** Comparison of performance of the representative catalysts with our catalysts^a^

Entry	Catalyst	Pd loading (ICP-AES)	H_2_O_2_ yield (mol/kg_cat_/h)	H_2_O_2_ yield (mol/g_Pd_/h)	H_2_O_2_ degradation (mol/kg_cat_/h)	H_2_ conversion (100%)	H_2_O_2_ Selectivity (100%)	Ref.
**Our results**								
Oxidized								
1	0.05%O-Pd/TiO_2_	0.05%	54	108	n.d.	1.84	> 99	–
2	0.1%O-Pd/TiO_2_	0.1%	115	115	n.d.	3.85	> 99	–
3	0.5%O-Pd/TiO_2_	0.5%	50	10	n.d.	4.31	40	–
4	1%O-Pd/TiO_2_	1%	79	7.9	n.d.	4.70	58	–
5	2%O-Pd/TiO_2_	1.5%	79	5.26	n.d.	5.44	50	–
6	3%O-Pd/TiO_2_−450	2.7%	49	1.81	n.d.	7.67	22	–
7	3%O-Pd/TiO_2_−600	2.7%	23	0.85	n.d.	11.32	7	–
Metallic								
8	0.1%M-Pd/TiO_2_	0.1%	n.d	n.d.	n.d.	n.d.	n.d.	
9	1%M-Pd/TiO_2_	0.8%	61	7.62	518	4.89	43	–
10	3%M-Pd/TiO_2_	2.7%	46	1.70	714	6.60	24	–
11	5%M-Pd/TiO_2_	4.6%	42	0.91	867	10.33	14	–
12	9%Pd/C	9%	30	0.33	1054	25.83	4	–
**Results in the literature**								
13	Pd_5_Zn/Al_2_O_3_	0.85%	216	25.431	177.54	56.6	78.5	^17^
14	0.5%Au/0.5%Pd/TiO_2_	0.5%	99	19.8	230	–	70	
15	Pd_6_Pb NRs/TiO_2_-H-A	3.16%	170.1	5.667	260	40	56.7	^5^
16	2.5%Au-2.5%Pd/C	2.5%	110	4.4	n.d.	–	80	^4^
17	3 wt%Pd–2 wt% Sn/TiO_2_	3%	61	2.03	n.d.	9	96	^23^
18	5 wt%Pd@NiO-3/TiO_2_	5%	89	1.78	8	–	91	^16^
19	2.5%Au–2.5%Pd/C	2.5%	160	6.4	n.d.	–	>98	^22^
20	Pd_1_Au_220_	0.011%	–	–	–	–	95 ± 3	^30^

^a^H_2_O_2_ yield was determined under the following reaction conditions: 5% H_2_/CO_2_ (3.0 MPa) and 25% O_2_/CO_2_ (1.2 MPa), 8.5 g solvent (2.9 g water, 5.6 g CH_3_OH), 2.5 mg catalyst, 2 °C, 1200 rpm, 30 min. H_2_O_2_ degradation was under standard reaction conditions: 5% H_2_/CO_2_ (3.0 MPa), 8.5 g solvent (5.6 g CH_3_OH, 2.34 g H_2_O, and 0.56 g 30% H_2_O_2_), 2.5 mg catalyst, 2 °C, 1200 rpm, 30 min. n. d., not detected. To ensure the reliability of the data, all the above experiments have to be tested for nine times, the data presented was the average value, the error of H_2_O_2_ yield and selectivity are within 1% and 4% respectively.

Although the performance of the O-Pd/TiO_2_ catalyst is superior to that of the M-Pd/TiO_2_, whether the oxidized Pd species possessing high activity and selectivity remain controversial. The Burch et al. propose that M-Pd species has a higher activity and selectivity to H_2_O_2_^8^. Conversely, the Choudhary and Gaikwad et al. showed that oxidized Pd species exhibit better catalytic activity and selectivity by loading M-Pd and PdO on different oxide supports^25,35^. H_2_ is easily activated on Pd^0^ sites, while O_2_ tends to be dissociated on successive Pd^0^ sites; however, O_2_ is stable on the surface of PdO^26^. Thus, both Pd^0^ and Pd^2+^ species play vital roles in H_2_O_2_ synthesis. Recently, the DFT calculation by Wang et al. suggested that PdO (101) can effectively inhibit the dissociation of O–O bonds, leading to better activity and selectivity than Pd (111)^27^, which is consistent with our experimental results.

**Fig. 4:**
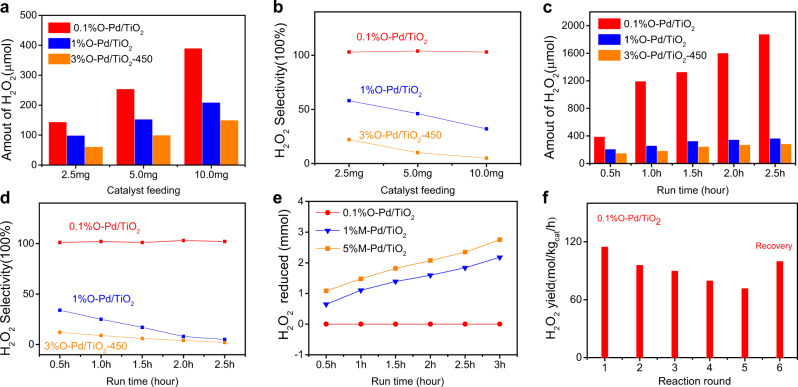
Catalytic performance. **a**, **b** The amounts of H_2_O_2_ and the H_2_O_2_ selectivity of different catalysts feeding. Reaction time: 30 min. **c**, **d** The amounts of H_2_O_2_ and the H_2_O_2_ selectivity of different reaction time. Catalyst feeding: 10 mg. **e** H_2_O_2_ degradation test. Reaction conditions: 5%H_2_/CO_2_ (3.0 Mpa), 2.5 mg of catalyst, 8.5 g solvent (2% H_2_O_2_),1200 rpm, 2 °C. **f** Stability test. Reaction conditions: 5%H_2_/CO_2_ (3.0 Mpa), 25%O_2_/CO_2_ (1.2 Mpa), 2.5 mg of catalyst, 8.5 g solvent, 1200 rpm, 2 °C, 30 min.

The influence of reaction conditions (catalyst feeding and reaction time) on catalytic performance were further studied. For 0.1%O-Pd/TiO_2_, the amount of H_2_O_2_ increases with the increase of catalyst feeding. For example, it can produce 144 μmol of H_2_O_2_ in half an hour when 2.5 mg 0.1%O-Pd/TiO_2_ catalysts are fed, 10 mg can produce 390 μmol of H_2_O_2_ (78 mol/kg_cat_/h), corresponding to 0.15% concentration (Fig. 4a), H_2_ conversion is 10.43%, which is also comparable to PdSn catalysts (9% of H_2_ conversion, 61 mol/kg_cat_/h, 96% of selectivity) (Table 1, Entry 17). It is also noteworthy that the H_2_O_2_ selectivity of 0.1%O-Pd/TiO_2_ is >99% regardless of the quantity of 2.5 mg, 5 mg, or 10 mg (Fig. 4b). On the contrary, for clusters and nanoparticles, the increase in the amount of H_2_O_2_ production is not obvious, but their H_2_O_2_ selectivity gradually decreases (Fig. 4a, b). Reaction time was extended from half an hour to three hours. We found that when the reaction time reached 2.5 h, the production of H_2_O_2_ was up to 1877 μmol (0.75% concentration) for 0.1%O-Pd/TiO_2_ (Fig. 4c). However, when the reaction time is more than 2.5 h, the concentration of H_2_O_2_ remains at 0.75%. The explanation might be that the large gas consumption in the reactor hinder the further generation of H_2_O_2_. To verify this point, we renew the gas in the reactor after the reaction of 2.5 h, and proceed with the reaction for the following 2.5 h (note the remaining gas in the reactor was completely discharged to 0 Mpa and then injected with 3.0 Mpa 5%H_2_/CO_2_ and 1.2 Mpa 25% O_2_/CO_2_). The results show that the concentration of H_2_O_2_ rose from 0.75% to 1.07% (2685 μmol). In general, H_2_O_2_ selectivity will decrease because of the side reactions and H_2_O_2_ degradation in a long-term reaction^8,23,24,31,32^. Interestingly, we found that the selectivity of 0.1%O-Pd/TiO_2_ is always >99% no matter the reaction time (Fig. 4d). But for clusters and nanoparticles, the H_2_O_2_ selectivity does decline (Fig. 4d). One interpretation of this phenomenon is that as H_2_ conversion increases, selectivity decreases due to H_2_O_2_ degradation. This can be better understood by comparing selectivity as a function of conversion for the different catalysts (Supplementary Fig. 5).

Therefore, we further measured the degradation rate of H_2_O_2_ under similar reaction conditions (3.0 Mpa 5%H_2_/CO_2_, the initial concentration of H_2_O_2_ is 2 wt.%). Firstly, the experiment shows that H_2_O_2_ does not degrade on TiO_2_ support itself at 2 °C (Supplementary Table 2), which is consistent with the results previously reported by Edwards et al.^22^. Interestingly, the H_2_O_2_ degradation rate was not detected on the O-Pd/TiO_2_ catalysts, but there was a significant H_2_O_2_ degradation rate on the M-Pd/TiO_2_ catalysts; with the increase of metallic Pd loading, the degradation rate of H_2_O_2_ becomes higher (Table 1, Supplementary Table 3). For example, the H_2_O_2_ degradation rate of 1% and 5%M-Pd/TiO_2_ were 518 and 867 mol/kg_cat_/h. To further understand the H_2_O_2_ degradation performance of catalysts, we have conducted a series of H_2_O_2_ degradation experiments under different conditions of atmosphere (5% H_2_/CO_2_ (3.0 MPa), 25% O_2_/CO_2_ (3.0 MPa) and pure N_2_ (3.0 MPa), 5%H_2_/N_2_ (3.0 Mpa). (Supplementary Table 4).

Under the 5% H_2_/CO_2_ (3.0 MPa), the degradation of H_2_O_2_ was not detected on O-Pd/TiO_2_ (Supplementary Table 4, Entry 1); to eliminate the influence of CO_2_, we used 5% H_2_/N_2_ for the degradation experiment, the degradation rate was still not detected (Supplementary Table 4, Entry 8); however, the amount of H_2_O_2_ gradually decreased with time for M-Pd/TiO_2_ catalyst. (Fig. 4e; Supplementary Table 4, Entry 2–3). On the other hand, we have not detected any degradation for both O-Pd/TiO_2_ and M-Pd/TiO_2_ catalyst under 25% O_2_/CO_2_ (3.0 MPa) or pure N_2_ (3 Mpa). (Supplementary Table 4, Entry 4–7), which indicates the slow degradation of H_2_O_2_ in the absence of H_2_. These observations suggested that the degradation rate of H_2_O_2_ is extremely low on the O-Pd/TiO_2_ catalyst even in the presence of H_2_. The result is meaningful for the degradation of H_2_O_2_ can be avoided without adding any inhibitors or pretreatment/post-treatment of catalysts.

Finally, we have investigated the stability of 0.1% O-Pd/TiO_2_ catalyst (Fig. 4f) by measuring the H_2_O_2_ yield in the cycle test. The H_2_O_2_ yield decreased from 115 to 74 mol/kg_cat_/h after reused five times. In the HAADF-STEM image of the used 0.1% O-Pd/TiO_2_ (Supplementary Fig. 6), we have observed some aggregated Pd species (about 3 nm Pd nanoparticle) on the TiO_2_ particle by using EDS mapping, however, the homogenous distribution of Pd species in other places suggest that many single Pd atom still existed. The XPS results of the used catalyst reveal the existence of oxidized Pd species other than the metallic Pd species (Supplementary Figs. 7 and 8), which is consistent with the STEM result. Since the metallic Pd species showed very poor H_2_O_2_ yield in the experiment (Table 1, Entry 9–11), the observed Pd aggregation might mainly account for the loss of H_2_O_2_ yield in the recycle test. The decrease of H_2_O_2_ yield in the recycle experiment further indicates that the performance of the catalyst after reaction (co-existence of a single Pd atom and Pd nanoparticles) is not as good as that of the fresh catalyst, highlighting the important role of the initial single Pd (2+) state.

To further confirm this hypothesis, we have heated the used catalyst (after 5 cycles) at 350 °C for 3 h in the air, and the H_2_O_2_ yield increase from 74 mol/kg_cat_/h to 100 mol/kg_cat_/h, which is close to the fresh catalyst (Fig. 4f, round 6). It was also reported that the anneal treatment in the oxidative atmosphere will re-disperse the noble nanoparticle to a single noble metal atom on the oxide support^36–38^. Thus, it’s reasonable to believe that the recovered H_2_O_2_ yield might be caused by the recovery of a single Pd^2+^ atom through annealing in the air, which also suggests the anneal treatment in the air is an effective method to refresh the used catalyst. The result again confirms that the single Pd^2+^ atom accounts for the high activity and selectivity towards the direct synthesis of H_2_O_2_.

### Density functional theory calculations

As mentioned in the introduction section, the H_2_O_2_ selectivity is mainly determined by the competitive reactions between *OOH formation and O–O bond cleavage on catalysts. For these reasons, first-principles calculations are used to investigate the dissociation of O_2_ and H_2_O_2_ over single atom and PdO clusters. Single Pd atom (Pd_1_/TiO_2_) and PdO clusters (Pd_8_O_8_/TiO_2_) structure models were optimized on the surface of TiO_2_ (101) (Supplementary Fig. 9). The Pd_1_/TiO_2_ model is based on the palladium-oxygen coordination number fitted by EXAFS, and Pd_8_O_8_ is extracted from the bulk phase of PdO, which simulates the coordination information of Pd and O in large nanoparticles. It is shown that O_2_ is adsorbed by “Pd-O-O” configuration on Pd_1_/TiO_2_ and is mainly adsorbed by “Pd-O-O-Pd” configuration on Pd_8_O_8_/TiO_2_ (Supplementary Fig. 10). The oxidation state of Pd single atom estimated by bader charge analysis is (+1.73) between +1 and +2 (Supplementary Fig. 11), which is consistent with the XAS results.

**Fig. 5:**
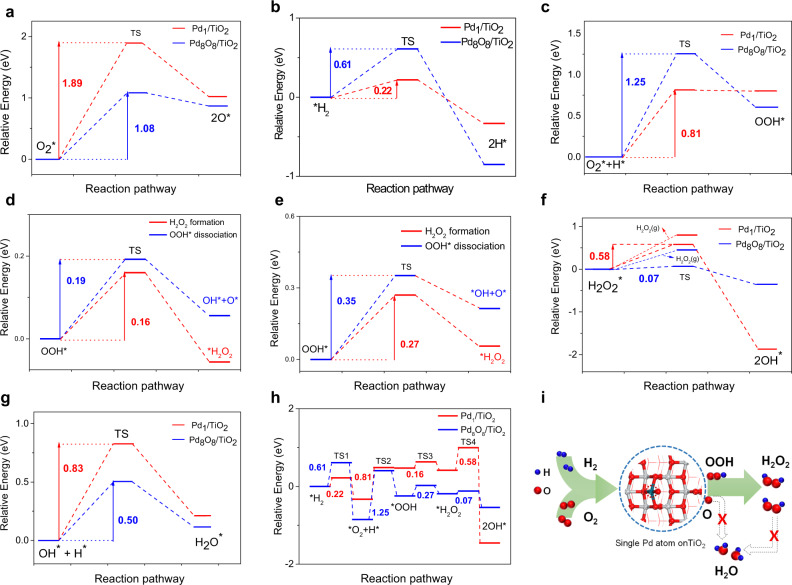
DFT calculations. **a** The reaction energy barriers of adsorbed *O_2_ dissociation steps on Pd_1_/TiO_2_ and Pd_8_O_8_/TiO_2_. **b** Energy profiles for H_2_ dissociation on Pd_1_/TiO_2_ and Pd_8_O_8_/TiO_2_. **c** The reaction energy barriers of adsorbed *O_2_ hydrogenation steps on Pd_1_/TiO_2_ and Pd_8_O_8_/TiO_2_. **d** The reaction energy barriers of H_2_O_2_ formation and OOH dissociation on Pd_1_/TiO_2_. **e** The reaction energy barriers of H_2_O_2_ formation and OOH dissociation on Pd_8_O_8_/TiO_2_. **f** The reaction energy barriers of adsorbed H_2_O_2_* dissociation steps on Pd_1_/TiO_2_ and Pd_8_O_8_/TiO_2_. **g** The reaction energy barriers of OH* with H* on Pd_1_/TiO_2_ and Pd_8_O_8_/TiO_2_. **h** The entire reaction potential energy landscape on Pd_1_/TiO_2_ and Pd_8_O_8_/TiO_2_. **i** Schematic illustration of H_2_O_2_ formation on single Pd atom catalyst.

Firstly, we have calculated the dissociation energy barrier of O_2_ on Pd_1_/TiO_2_ and Pd_8_O_8_/TiO_2_, respectively. The dissociation of O_2_ on Pd_1_/TiO_2_ is a high energy process that is endothermic by 1.89 eV (Fig. 5a), while the dissociation energy barrier of O_2_ on Pd_8_O_8_/TiO_2_ needs only 1.08 eV, indicating that the dissociation of O_2_ on Pd_1_/TiO_2_ is not favorable. And compared with the reported results of related DFT work (Supplementary Table 5), we find that the dissociation energy barrier of O–O bond on Pd_1_/TiO_2_ is the highest. This difference in the dissociation barrier can be attributed to structural differences (i.e., the adsorption configuration of oxygen on Pd). Oxygen can only be dissociated by migrating one oxygen atom to the nearby Ti site on Pd_1_/TiO_2_, whereas on Pd_8_O_8_/TiO_2_ it can be directly fractured by “Pd-O-O-Pd” (Supplementary Fig. 12). Moreover, we found that H_2_ is more easily activated on Pd_1_/TiO_2_ than Pd_8_O_8_/TiO_2_ (Fig. 5b).

Similarly, the formation of *OOH on Pd_1_/TiO_2_ only needs to overcome the energy barrier of 0.81 eV, while Pd_8_O_8_/TiO_2_ needs to overcome 1.25 eV (Fig. 5c). Structurally, the reason is that the adsorbed oxygen can directly capture the adsorbed hydrogen at the single Pd atom site, while clusters need to capture hydrogen from other Pd sites (Supplementary Fig. 13).

In addition, the reaction barriers for the step of OOH* hydrogenation to H_2_O_2_ and OOH* dissociation were investigated. On Pd_1_/TiO_2_, the formation of H_2_O_2_ only needs to cross an energy barrier of 0.16 eV, while OOH* dissociation needs to overcome 0.19 eV of barrier (Fig. 5d). In contrast, the formation of H_2_O_2_ needs to overcome 0.27 eV on Pd_8_O_8_/TiO_2_, OOH* dissociation needs to overcome 0.35 eV of barrier (Fig. 5e). The calculated results suggest that Pd_1_/TiO_2_ is much more favorable than Pd_8_O_8_/TiO_2_ for H_2_O_2_ formation.

The H_2_O_2_ degradation involved two steps (H_2_O_2_ → 2OH*; OH* + H* → H_2_O). The calculated energy barrier of H_2_O_2_ dissociation into 2OH* on Pd_1_/TiO_2_ (0.58 eV) is higher than that of Pd_8_O_8_/TiO_2_ (0.07 eV) (Fig. 5f), suggesting that H_2_O_2_ is more difficult to degrade on Pd_1_/TiO_2_. Moreover, the energy barrier of the reaction of the OH* with H* on Pd_1_/TiO_2_ (0.83 eV) is higher than that on the Pd_8_O_8_/TiO_2_ (0.50 eV) (Fig. 5g). Therefore, after the calculation of these transition states, the entire reaction potential energy landscape can be obtained (Fig. 5h). The results reveal that it is conducive to the generation of H_2_O_2_ on Pd_1_/TiO_2_ than on Pd_8_O_8_/TiO_2_, which is well consistent with the experimental observations. Therefore, a schematic diagram of H_2_O_2_ formation on single Pd atom catalyst is shown in Fig. 5i.

To sum up, the DFT calculation results confirm that single Pd atom catalysts favor the formation of the key intermediate (*OOH) and H_2_O_2_, but strongly suppress the cleavage of O − O bond in O_2_ and OOH and H_2_O_2_, leading to a higher activity and selectivity. The calculation results also show that the different performances come from various adsorption configurations of O_2_ on single atom and clusters at the beginning.

In summary, we have synthesized a series of O-Pd/TiO_2_ (oxidized) and M-Pd/TiO_2_ (metallic) catalysts for the oxygen hydrogenation to H_2_O_2_, the catalytic performance of O-Pd/TiO_2_ (including single Pd atom (0.1%), Pd cluster (1%)) are all better than that of M-Pd/TiO_2_ catalyst. Particularly, the single Pd atom catalyst displays ultrahigh activity (115 mol/g_Pd_/h), which is 14 and 135 times higher than that of clusters and nanoparticles, respectively; and the selectivity to H_2_O_2_ is more than 99%. More interesting, H_2_O_2_ degradation was also shut down. The concentration of H_2_O_2_ reached 1.07 wt.% in a batch. DFT calculations show that the O–O bond breaking is significantly inhibited on the single Pd atom and the O_2_ is easier to be activated to form *OOH and H_2_O_2_; and the energy barrier of H_2_O_2_ degradation is also higher. As a result, the high yield and selectivity is obtained on single Pd atom catalyst. The work reports the application of single Pd atom catalysts in the direct synthesis of H_2_O_2_. We believe it will yield far-reaching implications for subsequent catalyst design in direct synthesis of H_2_O_2_ and corresponding mechanism research in the future.

## Methods

### Materials

Titanium (IV) oxide, Aeroxide P25 (Beijing Balinwei Technology Co., Ltd, product of Japan), Ethylene glycol (A.R. Tianjin Damao Chemical Reagent Factory), Na_2_PdCl_4_ (>36.0%, Annege Chemical). Methyl alcohol (G.R. Tianjin Guangfu Science and Technology Development Co., Ltd). Fe (NH_4_)_2_·(SO_4_)_2_·6H_2_O (Tianjin Institute of Guangfu Fine Chemicals). Cerium sulfate (macklin reagent). Ultrapure water (18.2 MΩ cm). Stainless steel autoclave (Yanzheng Shanghai Instrument Co., Ltd). 5% H_2_/CO_2_, 5% H_2_/N_2_, pure N_2_ and 25% O_2_/CO_2_ were purchased from Beijing Millennium Capital Gas Co. Ltd.

### Synthesis of catalysts

Synthesis of O-Pd/TiO_2_ (oxidized): TiO_2_ was calcination at 450 °C for 4 h in the air (ramping rate: 5 °C/min) to remove surface water before catalysts preparation. The O-Pd/TiO_2_ catalyst was prepared using a hydrothermal synthesis method. The corresponding amount of Na_2_PdCl_4_ (10 g/L) was added to the TiO_2_ (1 g) carrier suspension and heated to 80 °C for 3 h under magnetic stirring. Then, the obtained Pd/TiO_2_ powder was centrifuged and freeze-dried overnight. Subsequently, the obtained catalyst was calcined at 350 °C for 3 h in the air at a heating rate of 5 °C/min. In order to obtain larger size palladium oxide nanoparticles, the samples with 3% palladium loading were heated at 450 °C and 600 °C for 3 h and other conditions remain unchanged. The prepared catalysts were denoted as 0.05%, 0.1%, 0.5%, 1%, 2% O-Pd/TiO_2_ respectively according to the Pd loading. The catalysts with 3% Pd loading were labeled as 3% O-Pd/TiO_2_−450 and 3% O-Pd/TiO_2_−600, respectively, according to the calcination temperature.

Synthesis of M-Pd/TiO_2_ (metallic): The preparation of M-Pd/TiO_2_ was like that of O-Pd/TiO_2_, except that the water was replaced with ethylene glycol and the temperature of TiO_2_ carrier suspension was 100 °C, under reflux conditions. The catalysts were not calcined with air after centrifugally freeze-dried and used directly. The prepared catalysts were marked as 0.1,1,3, and 5% M-Pd/TiO_2_ by the loading of palladium.

### Characterization

The Pd loading on the catalysts was analyzed using an IRIS Intrepid ER/S (Thermo Elemental) inductively coupled plasma-atomic emission spectrometer (ICP-AES). Transmission electron microscopy (TEM) images were obtained using a Tecnai F20 microscope in conjunction with powder samples deposited onto a copper micro-grid and coated with carbon, applying an accelerating voltage of 200 kV. Spherical aberration corrected (Cs corrected) high angle annular dark field scanning transmission electron microscopy (HAADF-STEM) and energy-dispersive X-ray (EDX) mapping images were obtained with an FEI Titan G2 microscope equipped with a Super-X detector, operating at 300 kV. X-ray diffraction (XRD) patterns of the samples were recorded on a Bruker D8 Advance system with Cu Kα radiation at 40 kV and 40 mA. X-ray photoelectron spectroscopy (XPS) analyses were performed using a Kratos Axis Ultra XPS spectrometer with monochromatized Al-Kα radiation and an energy resolution of 0.48 eV. The X-ray absorption fine structure (XAFS) spectrum data were collected on the BL14W1 beamline radiation equipment of the Shanghai Synchrotron Radiation Facility (SSRF) of the Shanghai Institute of Applied Physics (SINAP). Pd foil, PdO samples were used as references. All target samples and references were measured by fluorescence or transmission mode. Extended X-ray adsorption fine structure (EXAFS) fitting was conducted using the software of Artemis.

### Catalytic experimental measurement

Catalytic experiments were operated using a stainless-steel autoclave with a nominal volume of 50 mL and a maximum working pressure of 14 MPa.

H_2_O_2_ synthesis. In the typical experiment of H_2_O_2_ synthesis, 2.5 mg catalyst and 8.5 g solvent (5.6 g CH_3_OH (HPLC grade), 2.9 g H_2_O) were added into the autoclave. Before 3.0 MPa 5%H_2_/CO_2_ was injected at room temperature, the reactor was purged three times with 0.7 MPa (5%H_2_/CO_2_), then the temperature was reduced to 2 °C (in an ice bath), the pressure was about 2.3 MPa, at this time, 1.2 MPa 25% O_2_/CO_2_ was injected, and the total pressure was 3.5 MPa. Temperature and pressure are respectively detected by thermocouple and pressure sensor. The stirring speed was controlled at 1200 rpm.

The H_2_O_2_ yield was detected by acidified Ce (SO_4_)_2_ (0.01 M) titration in the presence of two drops of ferroin indicator. The Ce (SO_4_)_2_ solutions were standardized against (NH_4_)_2_Fe(SO_4_)_2_·6H_2_O using ferroin as an indicator. Catalyst yields are marked as mol H_2_O_2_ kg_cat_^−1 ^h^−1^ according to the following equation.

1 $${{{{{{\rm{H}}}}}}}_{2}{{{{{{\rm{O}}}}}}}_{2}\,{{{{{\rm{Yield}}}}}}=({{{{{\rm{moles}}}}}}\,{{{{{\rm{of}}}}}}\,{{{{{{\rm{H}}}}}}}_{2}{{{{{{\rm{O}}}}}}}_{2}\,{{{{{\rm{generated}}}}}})/({{{{{\rm{mass}}}}}}\,{{{{{\rm{of}}}}}}\,{{{{{\rm{catalyst}}}}}}\times {{{{{\rm{time}}}}}})$$

Gas analysis was performed by gas chromatography (GC-2020) equipped with a TDX-01 column connected to a thermal conductivity detector. Conversion of H_2_ was calculated by gas analysis before and after the reaction. H_2_O_2_ selectivity was calculated according to the following equation:

2 $${{{{{{\rm{H}}}}}}}_{2}{{{{{{\rm{O}}}}}}}_{2}\,{{{{{\rm{Selectivity}}}}}}=({{{{{\rm{moles}}}}}}\,{{{{{\rm{of}}}}}}\,{{{{{{\rm{H}}}}}}}_{2}{{{{{{\rm{O}}}}}}}_{2}\,{{{{{\rm{generated}}}}}})/({{{{{\rm{mass\; of}}}}}}\,{{{{{{\rm{H}}}}}}}_{2}{{{{{\rm{reacted}}}}}})\times 100\%$$

H_2_O_2_ degradation. The experiments were manipulated in a similar way to the H_2_O_2_ synthesis, but in the absence of 1.2 MPa 25%O_2_/CO_2_. In detail, H_2_O from the 8.5 g of solvent was replaced by a 30% H_2_O_2_ solution to give a reaction solvent containing between 2-8 wt%H_2_O_2_. The standard reaction conditions adopted for H_2_O_2_ degradation were as follows: 2.5 mg catalyst, 8.5 g solvent (5.6 g CH_3_OH, 2.34 g H_2_O, and 0.56 g 30% H_2_O_2_, 3.0 MPa 5%H_2_/CO_2_, 2 °C, 1200 rpm, 30 min). The H_2_O_2_ degradation rate is a combination of H_2_O_2_ hydrogenation and decomposition. H_2_O_2_ degradation rate was calculated following:

3 $${{{{{{\rm{H}}}}}}}_{2}{{{{{{\rm{O}}}}}}}_{2}\,{{{{{\rm{degradation\; rate}}}}}}=({{{{{\rm{moles}}}}}}\,{{{{{\rm{of}}}}}}\,{{{{{{\rm{H}}}}}}}_{2}{{{{{{\rm{O}}}}}}}_{2}\,{{{{{\rm{reduced}}}}}})/({{{{{\rm{mass}}}}}}\,{{{{{\rm{of}}}}}}\,{{{{{\rm{catalyst}}}}}}\times {{{{{\rm{time}}}}}})$$

To ensure the reliability of the data, all the above experiments have to be tested for nine times, the data presented was the average value, the error of H_2_O_2_ yield and selectivity are within 1%, 4% respectively.

### Calculation details

All the spin-polarized density functional (DFT) calculations were carried out with the Vienna Ab initio Simulation Package(VASP)^39^. The ion-electron interaction was described by the projector augmented wave (PAW) method^40^. The generalized gradient approximation (GGA) in the Perdew-Burke-Ernzerhof (PBE) functional was used for the exchange-correlation interactions^41^. The DFT-D3 method was introduced to describe van der Waals interactions^42^. The 15 Å vacuum slab in the z direction was applied to avoid interactions with adjacent units. The cut-off energy for plane-wave basis set was 450 eV. The convergence criterion for energy and force were set as 10-5 eV and 0.03 eV/Å during geometry optimization, respectively. The Brillouin zone was sampled with 2 × 2 × 1 k-point grids. Transition states were located with the climbing-image nudged elastic band (CI-NEB) method^43^, and the threshold value for the forces on each atom was 0.05 eV/Å.

## Supplementary information

Supplementary Information

Peer Review File

## Data Availability

The data supporting this study are available within the paper and the Supplementary Information. All other relevant source data are available from the corresponding authors upon reasonable request. Source data are provided with this paper.

## Source data

Source Data
