# Supplementary material for: High activity and selectivity of single palladium atom for oxygen hydrogenation to H2O2
Source: Nat Commun. 2022 Aug 12;13:4737. doi: 10.1038/s41467-022-32450-6 (PMC9374736; doi:10.1038/s41467-022-32450-6)
Supplement: Supplementary file 1 — Supplementary Information [file 41467_2022_32450_MOESM1_ESM.pdf]

Supplementary Information

High activity and selectivity of single palladium atom for oxygen hydrogenation to H_2O_2

Shiming Yu[1], Xing Cheng[1],Yueshuai Wang[2], Bo Xiao[1], Yiran Xing[1], Jun Ren[3],Yue Lu[2], Hongyi Li[2]*, Chunqiang Zhuang[2]*, Ge Chen[1]*

[1]:Beijing Key Laboratory for Green Catalysis and Separation, Faculty of Environment and Life, Beijing University of Technology, Beijing 100124, P. R. China.

[2]: Faculty of Materials and Manufacturing, Beijing University of Technology.

[3]: North University of China, Taiyuan, 030051, P. R. China.

E-mail of corresponding author: lhy06@bjut.edu.cn;chunqiang.zhuang@bjut.edu.cn; chenge@bjut.edu.cn

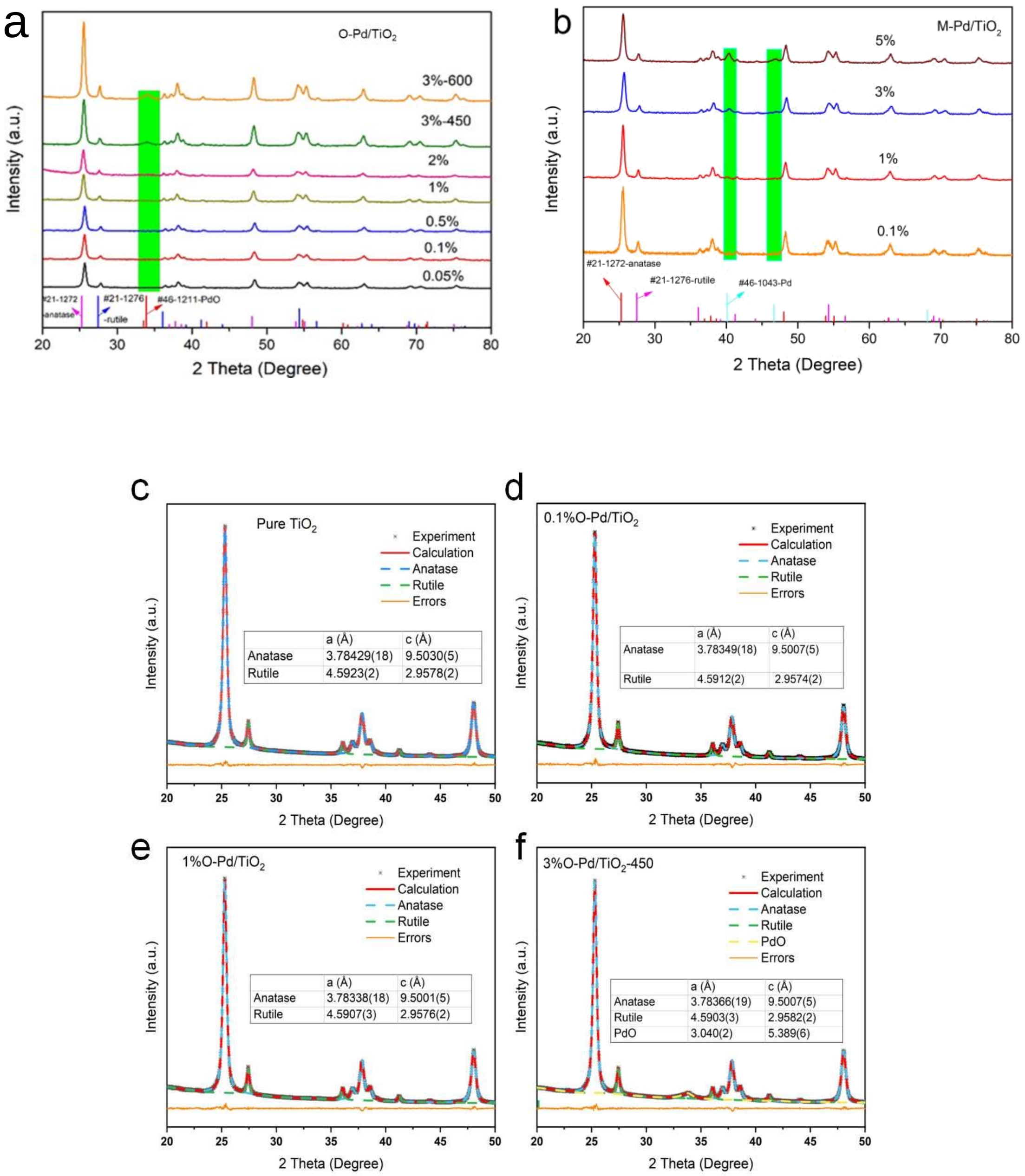

Supplementary Figure 1. XRD characterization of the catalysts. (a) O-Pd/TiO_2 and (b) M-Pd/TiO_2, (c-f) XRD refinements of different catalysts

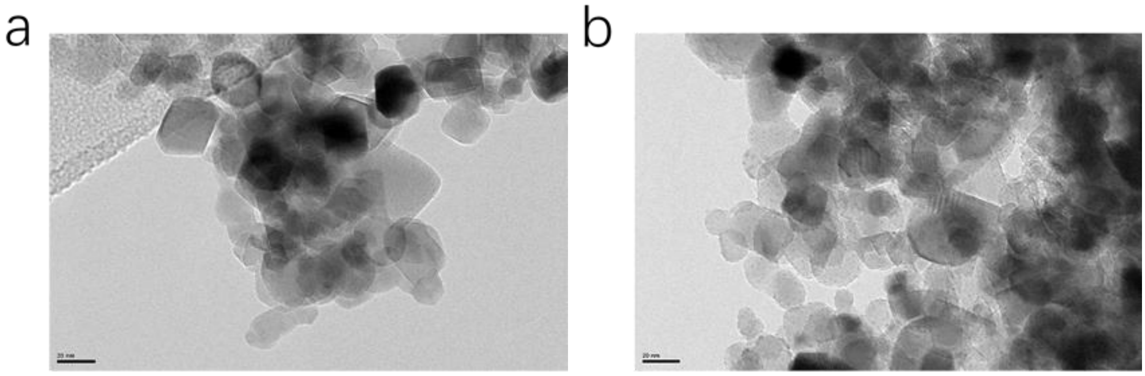

Supplementary Figure 2. Electron microscopic characterization of catalyst. TEM of different catalysts with 20 nm scale bars: (a) 0.1%O-Pd/TiO_2 (b)1%O-Pd/TiO_2

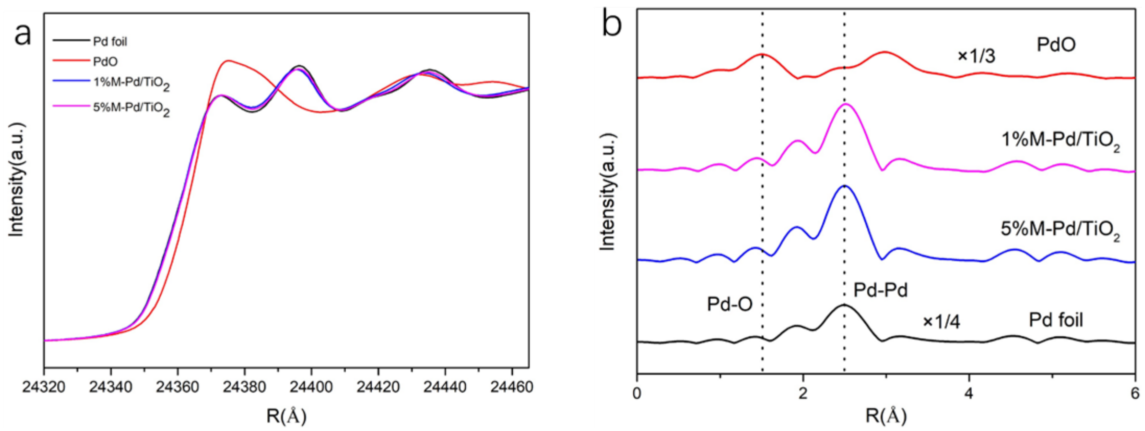

Supplementary Figure 3. X-ray absorption spectrum of catalyst. (a) XANES spectra at the Pd K-edge and (b) the k^2-weighted Fourier transforms of Pd K-edge EXAFS spectra for M-Pd/TiO_2, PdO,and Pd foil.

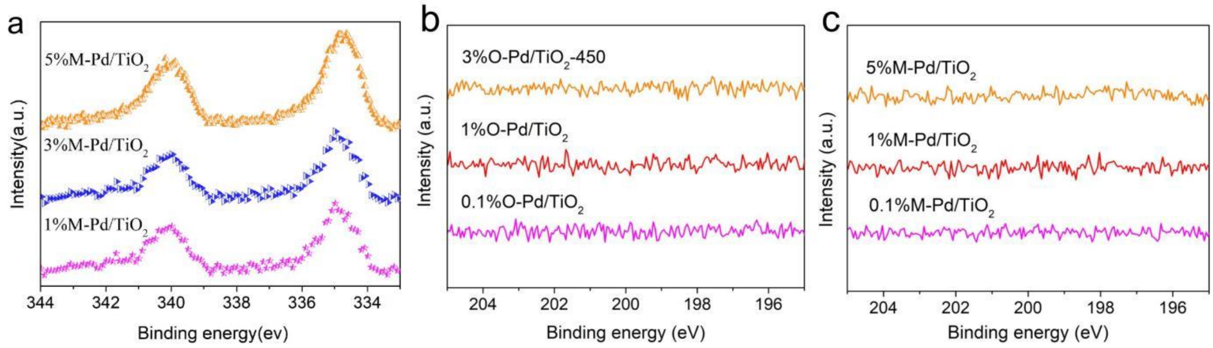

Supplementary Figure 4. The X-ray photoelectron spectra of catalysts. (a) Pd 3*d*. (b) and (c) Cl 2*p*

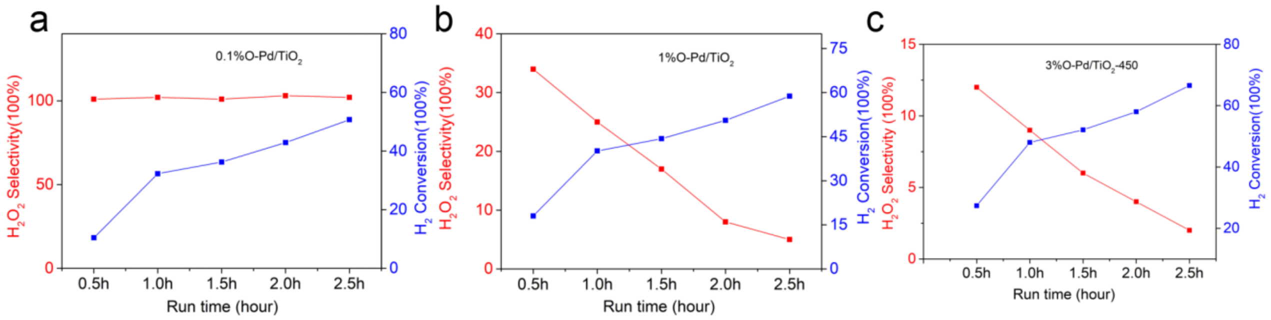

Supplementary Figure 5. H_2 conversion and H_2O_2 selectivity with different reaction time on various loading catalysts (catalyst feeding: 10 mg). (a) 0.1%O-Pd/TiO_2. (b) 1%O-Pd/TiO_2. (c) 3%O-Pd/TiO_2-450.

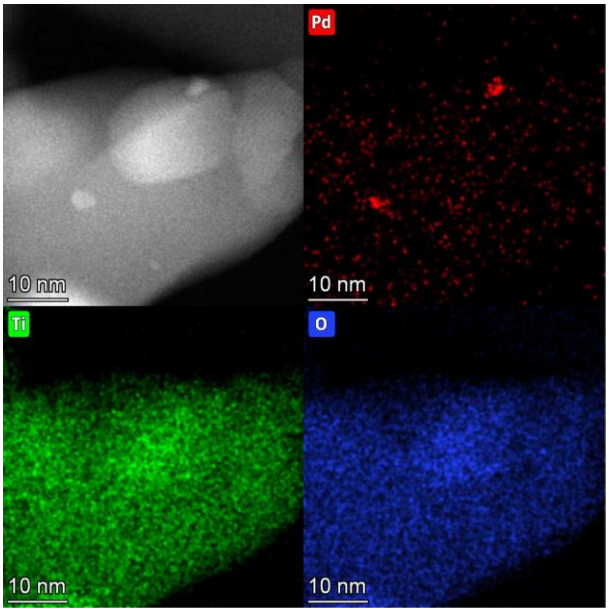

Supplementary Figure 6. STEM Characterization of catalyst after reaction. The HAADF-STEM image of the used 0.1%O-Pd/TiO_2 catalysts with 10 nm scale bars.

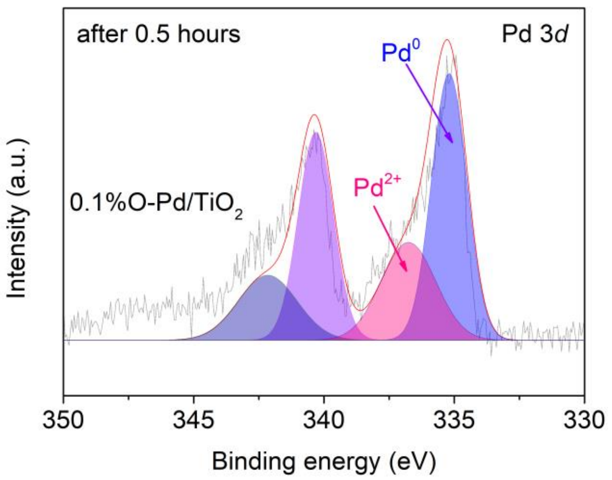

Supplementary Figure 7. XPS Characterization of catalyst after reaction. Pd 3*d* XPS spectra of 0.1%O-Pd/TiO_2 catalysts after 0.5 hours reactions.

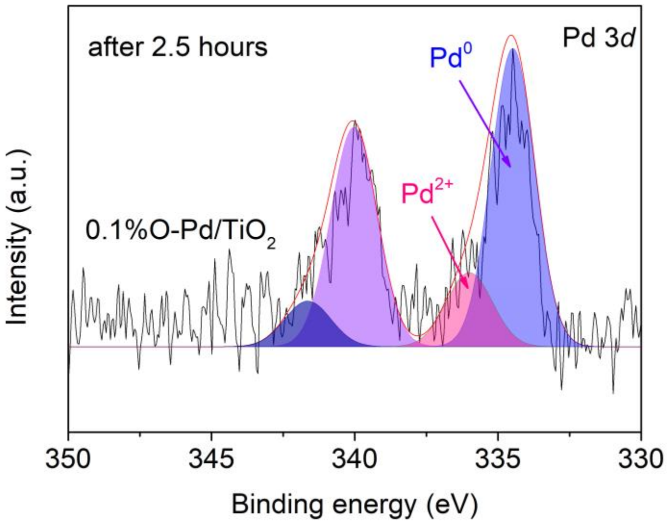

Supplementary Figure 8. XPS Characterization of catalyst after reaction. Pd 3*d* XPS spectra of 0.1%O-Pd/TiO_2 catalysts after 2.5 hours reactions.

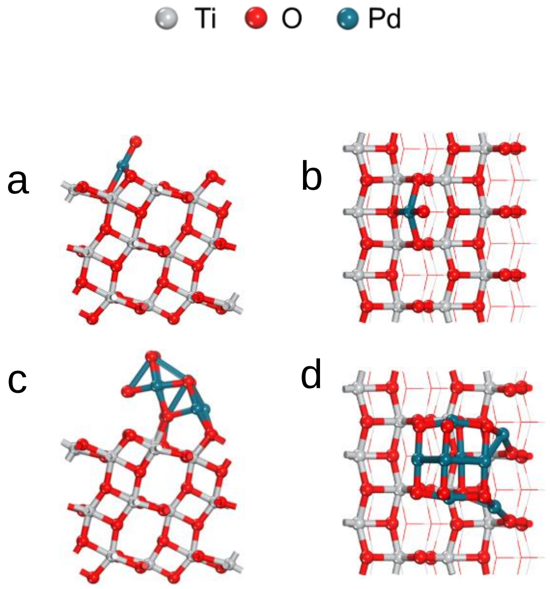

Supplementary Figure 9. Models of single atom and cluster. (a) Side view (b) Top view of Pd_1/TiO_2 (c) Side view (d) Top view of Pd_8O_8/TiO_2

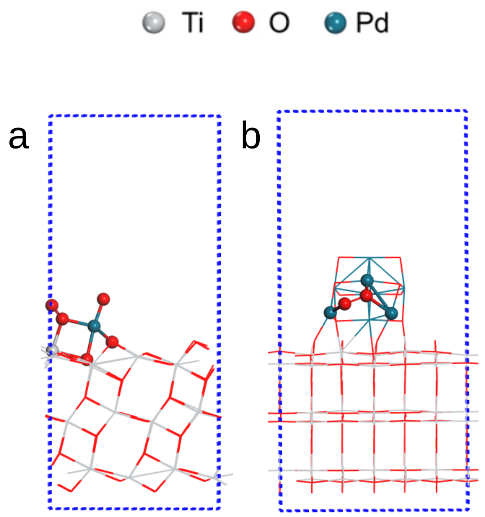

Supplementary Figure 10. O_2 adsorption configuration after structure optimization. (a).Pd_1/TiO_2,(b).Pd_8O_8/TiO_2

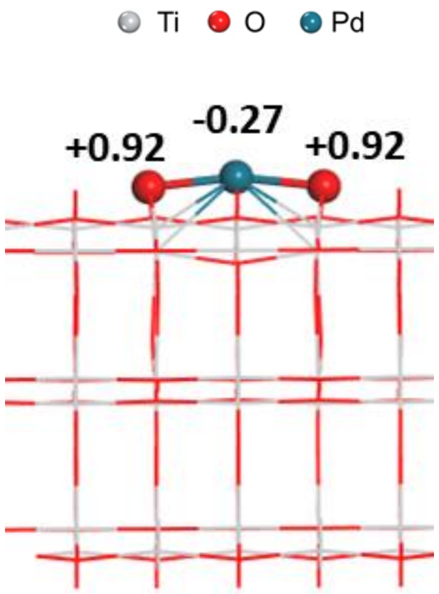

Supplementary Figure 11. Bader charge analysis. The oxidation state of Pd single atom estimated by bader charge analysis.

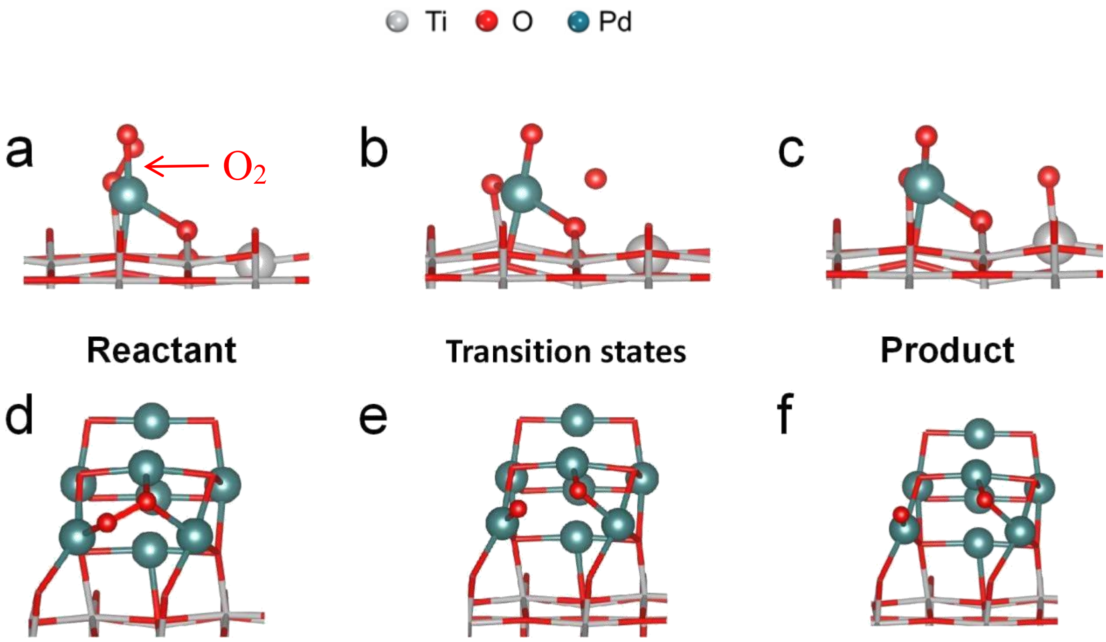

Supplementary Figure 12. The structural configurations of adsorbed *O_2 dissociation steps. (a,b,c) Pd_1/TiO_2 and (d,e,f) Pd_8O_8/TiO_2.

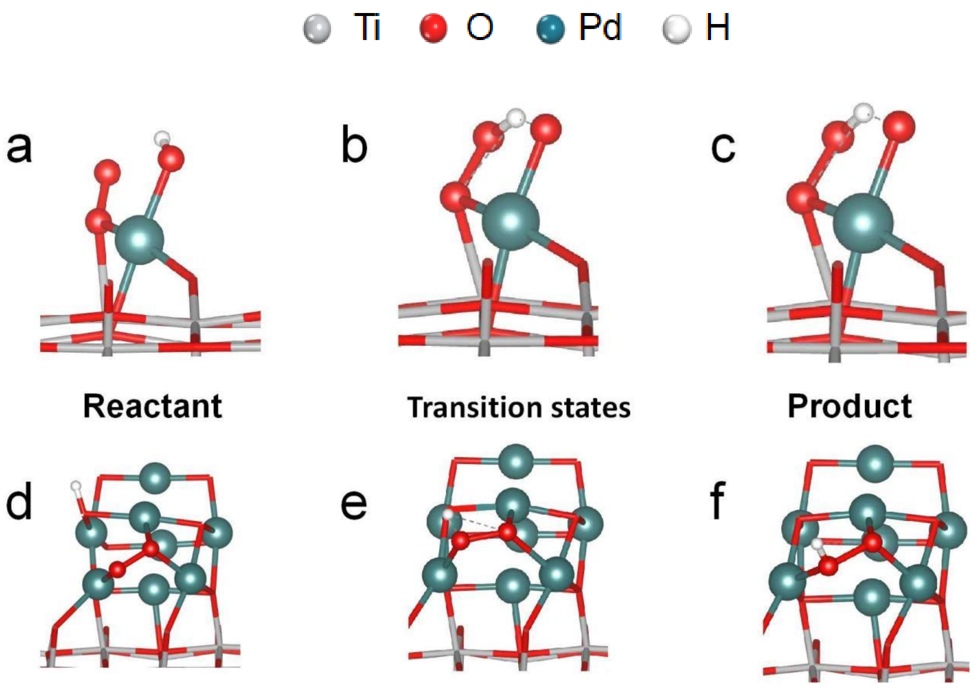

Supplementary Figure 13. The structural configurations of adsorbed adsorbed $*O_2$ hydrogenation to $OOH*$ steps. (a,b,c) Pd_1/TiO_2 and (d,e,f) Pd_8O_8/TiO_2.

Supplementary Table 1. EXAFS fitting parameters at the Pd K-edge for various samples (S_0^2=0.80)[a]

	shell	CN	R(Å)	σ^2	ΔE_0	R factor
Pd foil	Pd-Pd	12	2.74±0.01	0.0056	-3.7±0.5	0.0051
PdO	Pd-O	4.2±0.2	2.02±0.01	0.0016	3.1±1.0	0.0066
	Pd-Pd	4.5±0.3	3.06±0.01	0.0037		
	Pd-Pd1	6.9±0.4	3.44±0.01			
0.1%O-Pd/TiO_2	Pd-O	3.8±0.2	2.04±0.01	0.0049	6.3±1.7	0.0064
1% O-Pd/TiO_2	Pd-O	4.3±0.2	2.04±0.01	0.0039	5.8±1.4	0.0154
	Pd-Pd	1.3±0.2	3.04±0.01	0.0013		
	Pd-Pd1	1.2±0.3	3.49±0.02			
3% O-Pd/TiO_2-450	Pd-O	4.2±0.2	2.03±0.01	0.0033	4.2±0.9	0.0094
	Pd-Pd	3.8±0.3	3.06±0.01	0.0061		
	Pd-Pd1	3.8±0.4	3.46±0.01			

a: CN: coordination numbers; b: R: bond distance; c: σ^2: Debye-Waller factors; d: ΔE_0: the inner potential correction. R factor: goodness of fit. S_0^2 was set to 0.80, according to the experimental EXAFS fit of Pd foil reference by fixing CN as the known crystallographic value; δ: percentage.

Supplementary Table 2. H_2O_2 degradation test on TiO_2 support itself [a]

	H_2O_2 degradation (mol/kg$_{cat}$/h)					
TiO_2	0.5 h	1.0 h	1.5 h	2.0 h	2.5 h	3.0 h
	n.d	n.d	n.d	n.d	n.d	n.d

a: H_2O_2 degradation was under standard reaction conditions: 5% H_2/CO_2 (3.0 MPa), 8.5 g solvent (2% H_2O_2-8%H_2O_2), 2.5-10 mg TiO_2, 2°C, 1200 rpm, reaction time: 0.5 hour to 3 hours. n.d=not detected. 2% H_2O_2(5.6 g CH_3OH,2.34 g H_2O, and 0.56 g 30% H_2O_2).4% H_2O_2(5.6g CH_3OH,1.77 g H_2O, and 1.13 g 30% H_2O_2); 8% H_2O_2(5.6g CH_3OH,0.63 g H_2O, and 2.27 g 30% H_2O_2).

Supplementary Table 3. H_2O_2 degradation test on various catalysts.[a]

Entry	Catalyst	2%H_2O_2 (mol/kg$_{cat}$/h)	4%H_2O_2 (mol/kg$_{cat}$/h)	8%H_2O_2 (mol/kg$_{cat}$/h)
1	1%M-Pd/TiO_2	518	688	850
2	3%M-Pd/TiO_2	714	872	969
3	5%M-Pd/TiO_2	867	1024	1130
4	9%Pd/C	1054	1215	1292
5	0.05%-3%O-Pd/TiO_2	n.d.	n.d.	n.d.

a: H_2O_2 degradation was under standard reaction conditions: 5% H_2/CO_2 (3.0 MPa), 8.5 g solvent, 2.5 mg catalyst, 2°C, 1200 rpm, 30 min. n. d., not detected. 2% H_2O_2(5.6 g CH_3OH,2.34 g H_2O, and 0.56 g 30% H_2O_2).4% H_2O_2(5.6g CH_3OH,1.77 g H_2O, and 1.13 g 30% H_2O_2); 8% H_2O_2(5.6g CH_3OH,0.63 g H_2O, and 2.27 g 30% H_2O_2).

Supplementary Table 4. H_2O_2 degradation test under different atmospheres.[a]

Entry	Catalyst	H_2O_2 degradation (mol/kg$_{cat}$/h)					
		0.5 h	1.0 h	1.5 h	2.0 h	2.5 h	3.0 h
1	0.1%O-Pd/TiO_2[a]	n.d	n.d	n.d	n.d	n.d	n.d
2	1%M-Pd/TiO_2[a]	518	442	371	319	294	291
3	5%M-Pd/TiO_2[a]	867	590	484	414	376	367
4	0.1%O-Pd/TiO_2[b]	n.d	n.d	n.d	n.d	n.d	n.d
5	1%M-Pd/TiO_2[b]	n.d	n.d	n.d	n.d	n.d	n.d
6	0.1%O-Pd/TiO_2[c]	n.d	n.d	n.d	n.d	n.d	n.d
7	1%M-Pd/TiO_2[c]	n.d	n.d	n.d	n.d	n.d	n.d
8	0.1%O-Pd/TiO_2[d]	n.d	n.d	n.d	n.d	n.d	n.d

a: 5% H_2/CO_2 (3.0 MPa), b: 25% O_2/CO_2 (3.0 MPa).c: pure N_2 (3.0 MPa), d:5%H_2/N_2 ,8.5 g solvent (5.6 g CH_3OH, 2.34 g H_2O, and 0.56 g 30% H_2O_2), 2.5 mg catalyst, 2°C, 1200 rpm. n. d., not detected.

Supplementary Table 5. A summary of some relevant DFT work.

Entry	Refence	$*O_2 \rightarrow 2O*$	$*O_2+H* \rightarrow OOH*$	$*OOH+*H \rightarrow H_2O_2*$	$*OOH \rightarrow *OH+O*$
1	Pd(111) [1]	0.70 eV	0.68 eV	0.58 eV	0.11 eV
2	PdO(101) [1]	1.81 eV	0.03 eV	0.46 eV	1.33 eV
3	Pd(111) [2]	0.78 eV	0.58 eV	0.41 eV	0.33 eV
4	Pd(100) [2]	0.16 eV	0.67 eV	0.44 eV	0.26 eV
5	Pd(111) [3]	0.63 eV	0.54 eV	0.86 eV	0.11 eV
6	β-PdH(111)[3]	1.12 eV	0.22 eV	0.17 eV	0.34 eV
7	This work, Pd_1/TiO_2	1.89 eV	0.81 eV	0.16 eV	0.19 eV
8	This work, Pd_8O_8/TiO_2	1.08 eV	1.25 eV	0.27 eV	0.35 eV

Note : Compared with the reported results of related DFT works, we find that the dissociation energy barrier of O-O bond on Pd_1/TiO_2 (1.89eV) is the highest. In fact, it is controversial to compare the calculation results with different DFT literatures, because their calculation parameters and calculation system are different. However, the comparison is also useful for reference.

Supplementary References

[1] Fang Wang. et al. How Does the Oxidation State of Palladium Surfaces Affect the Reactivity and Selectivity of Direct Synthesis of Hydrogen Peroxide from Hydrogen and Oxygen Gases? A Density Functional Study. J. Am. Chem. Soc. 141, 901-910 (2019).

[2] Pengfei Tian. et al. Density functional theory study of direct synthesis of H_2O_2 from H_2 and O_2 on Pd (111), Pd (100), and Pd (110) surfaces. Chinese Journal of Catalysis. 34, 1002-1012 (2013).

[3] Lin Chen. et al. On the Reaction Mechanism of Direct H_2O_2 Formation over Pd Catalysts. ACS Catalysis. 11, 2735-2745 (2021).
